# Effect of *Ex Vivo* Culture Conditions on Immunosuppression by Human Mesenchymal Stem Cells

**DOI:** 10.1155/2013/154919

**Published:** 2013-06-04

**Authors:** Myoung Woo Lee, Dae Seong Kim, Somi Ryu, In Keun Jang, Hye Jin Kim, Jin Mo Yang, Doo-Hoon Lee, Soo Hyun Lee, Meong Hi Son, Hee Won Cheuh, Hye Lim Jung, Keon Hee Yoo, Ki Woong Sung, Hong Hoe Koo

**Affiliations:** ^1^Department of Pediatrics, Samsung Medical Center, Sungkyunkwan University College of Medicine, 50 Irwon-dong, Gangnam-gu, Seoul 135-710, Republic of Korea; ^2^Biomedical Research Institute, LIFELIVER. Co., LTD., 877-3 Jukjeon-dong, Suji-gu, Yongin-si, Gyeonggi-do 448-807, Republic of Korea; ^3^Department of Pediatrics, Dong-A Medical Center, Dong-A University College of Medicine, 26 Daesingongwonro, Soe-gu, Busan 602-172, Republic of Korea

## Abstract

A microarray analysis was performed to investigate whether *ex vivo* culture conditions affect the characteristics of MSCs. Gene expression profiles were mainly influenced by the level of cell confluence rather than initial seeding density. The analysis showed that 276 genes were upregulated and 230 genes downregulated in MSCs harvested at ~90% versus ~50% confluence (*P* < 0.05, FC > 2). The genes that were highly expressed in MSCs largely corresponded to chemotaxis, inflammation, and immune responses, indicating direct or indirect involvement in immunomodulatory functions. Specifically, PTGES and ULBP1 were up-regulated in MSCs harvested at high density. Treatment of MSCs with *PTGES* or *ULBP1* siRNA reversed their inhibition of T-cell proliferation *in vitro*. The culture conditions such as cell confluence at harvest seem to be important for gene expression profile of MSCs; therefore, the results of this study may provide useful guidelines for the harvest of MSCs that can appropriately suppress the immune response.

## 1. Introduction

The International Society for Cellular Therapy provided three minimal criteria to define human mesenchymal stem cells (MSCs) with regards to their characteristics of culture, biomarkers, and developmental potential [1]. First, MSCs must be plastic-adherent when maintained in standard culture conditions. Second, MSCs must express CD105 (SH2), CD73 (SH3/4), and CD90 and must not express CD45, CD34, CD14 CD11b, CD79*α*, CD19, or HLA-DR. Third, MSCs must differentiate into osteoblasts, adipocytes, and chondroblasts *in vitro*. Human MSCs can be isolated from a wide variety of tissues [2] and are promising candidates for cell-based transplantation and regenerative medicine therapies. Some of the unique features of MSCs that make them attractive targets for therapeutic applications are their tendency to preferentially home to damaged tissues, their unique immunosuppressive properties [3], their capacity for self-renewal, and their multilineage differentiation potential [4].

However, the morphology, proliferation rate, secreted factors, and characteristics of MSCs are heterogeneous due to their isolation methods, culture conditions, and senescent state, and this heterogeneity is difficult to molecularly characterize by traditional methods [2–7]. For example, differences in the number of passages that MSCs can be cultured for might be due to differences in culture techniques; however, the doubling potential of MSCs also varies, and it is impossible to predict when MSCs are close to becoming senescent. Cellular senescence is affected by the following factors: (1) anatomical site from which MSCs are derived; (2) age of the donor from which MSCs are derived; (3) growth media and serum supplements; (4) biomaterials; (5) cell culture technique [8]. This naturally existing cellular heterogeneity presents a challenge with regards to the molecular characterization of MSCs to improve their therapeutic value.

Several previous studies suggest that low initial plating densities are beneficial for optimal *ex vivo* expansion of MSCs and their subsequent differentiation [9–11]. A number of preclinical and clinical trials of MSCs are currently in progress worldwide; however, protocols for the use of MSCs, including optimized culture conditions, have not been standardized [7, 12]. Thus, complications in the *ex vivo* expansion of MSCs and the aforementioned heterogeneity may be important factors leading to the failure of *in vivo* experiments.

In this study, we used DNA microarray technology to examine the effects of the initial cell density and the cell density at harvesting on the gene expression of MSCs and the influence of these effects on the immunosuppressive properties of MSCs. The results of this study may provide useful guidelines for the collection of MSCs that can be used to appropriately modulate the immune response for the treatment of immune-related disorders. 

## 2. Materials and Methods

### 2.1. Isolation and Culture of Human MSCs

The Institutional Review Board of Samsung Medical Center approved this study (IRB File no. SMC 2011-06-084-001), and all samples (iliac crest bone marrow aspirates) were obtained from healthy adult volunteers who provided informed consent (2 samples, 42-year-old male and 35-year-old male). Mononuclear cells were isolated from bone marrow (BM) aspirates using density gradient centrifugation (Histopaque-1077; Sigma-Aldrich, St. Louis, MO). Cells were plated at a density of 3 × 10^5^ cells/cm^2^ in low glucose Dulbecco's Modified Eagle's Medium (LG-DMEM; Invitrogen-Gibco, Rockville, MD) containing 10% fetal bovine serum (FBS; Invitrogen-Gibco) and 100 U/mL penicillin/streptomycin (Invitrogen-Gibco). After 24 h, nonadherent cells were removed, and adherent cells were cultured for an additional 5–10 d or until they reached ~70% confluence. In this study, culture condition 1 (CC1) refers to a culture condition with an initial cell density of 200 cells/cm^2^ and a culture duration of 7 d, whereas CC2 and CC3 refer to 5000 cells/cm^2^ in 2 d and 7 d cultures, respectively. The medium was changed every 2 to 3 d. Confluence was estimated by phase microscopy (Olympus CK-40; Olympus Cor., Tokyo, Japan) based on the occupied surface of the tissue culture flask, and MSC density was determined by microscopic enumeration with a hematocytometer (Marienfeld, Germany). 

### 2.2. Immunophenotypic Analysis

Primary antibodies against human antigen CD73, CD90, CD105, CD166, CD14, CD34, CD45, and histocompatibility antigen DR alpha chain (HLA-DR) were purchased from BD Biosciences (San Jose, CA). MSCs (5 × 10^5^ cells) were resuspended in 500 *μ*L phosphate-buffered saline (PBS; Invitrogen-Gibco) and cultured with fluorescein-isothiocyanate- (FITC-) or phycoerythrin- (PE-) conjugated primary antibodies for 20 min at room temperature (RT). FITC- or PE-conjugated human IgGs were used as isotype controls at the same concentration as the specific primary antibodies. The fluorescence intensity of the cells was evaluated by flow cytometry (FACScan; BD Biosciences). Data were analyzed using the CELLQUEST software (BD Biosciences).

### 2.3. Differentiation Assays

#### 2.3.1. Osteogenic Induction

 Upon reaching 50% confluency, cells were cultured for 14–21 days in LG-DMEM containing 10% FBS, 0.1 *μ*M dexamethasone (Sigma-Aldrich), 10 mM *β*-glycerophosphate (Sigma-Aldrich), and 100 *μ*M ascorbate-2-phosphate (Sigma-Aldrich). The medium was changed every 3 days. Osteogenic differentiation was confirmed by the expression of alkaline phosphatase.

#### 2.3.2. Adipogenic Induction

Upon reaching 100% confluency, cells were cultured for 14–21 days in LG-DMEM containing 10% FBS, 1 *μ*M dexamethasone, 0.5 *μ*M isobutyl methylxanthine (Sigma-Aldrich), 100 *μ*M indomethacin (Sigma-Aldrich), and 10 *μ*g/mL insulin (Sigma-Aldrich). Adipogenic differentiation was evaluated by the cellular accumulation of neutral lipid vacuoles that were stained with Oil Red O (Sigma-Aldrich). 

#### 2.3.3. Chondrogenic Induction: Pellet Assay

 Upon reaching 80% confluency, cells were trypsinized with 0.05% trypsin-EDTA and resuspended in LG-DMEM containing 1× insulin-transferrin-selenium (ITS; Invitrogen-Gibco), 1 mM sodium pyruvate (Invitrogen-Gibco), 0.1 *μ*M dexamethasone, 397 *μ*g/mL ascorbate-2-phosphate, and 10 ng/mL transforming growth factor- (TGF-) *β*_1_ (R&D Systems, Minneapolis, MN). Viable cells were counted and seeded at a density of 2 × 10^5^ cells per tube in 15 cm^3^ conical tubes. Cells were collected at the bottom of the tubes by centrifugation, and compact cell pellets were allowed to form in a humidified atmosphere at 37°C with 5% CO_2_. After 14–21 days of culture, chondrogenic differentiation was detected by the extracellular accumulation of chondrocyte matrix that was stained with toluidine blue (Sigma-Aldrich).

### 2.4. RNA Isolation and Microarray Analysis

Total cellular RNA was extracted using TRIzol (Invitrogen-Gibco) and purified using an RNeasy column (Qiagen, Valencia, CA). RNA quality was determined by denaturing gel electrophoresis, the OD 260/280 ratio, and analysis on an Agilent 2100 Bioanalyzer (Agilent Technologies, Palo Alto, CA). Biotinylated complementary RNA was prepared and hybridized to the Illumina Human HT-12 Expression BeadChip (Illumina, San Diego, CA). The arrays were scanned and analyzed with Illumina Genome Studio v2009.2 software (Gene Expression Module v1.5.4, Illumina). The false discovery rate was controlled by adjusting *P* values using the Benjamini-Hochberg algorithm, followed by performance of Gene Set Enrichment Analysis and a one-tailed Fisher's exact test. Microarray data of 24,526 probes were filtered by applying two criteria for significance: *P* < 0.05 and fold change (FC) > 2.

### 2.5. Quantitative Real-Time PCR (qRT-PCR) Analysis

CDNA was produced using the Superscript RT-PCR System (Invitrogen, Karlsruhe, Germany) according to the manufacturer's recommendations for oligo (dT)_20_-primed cDNA synthesis. QRT-PCR was performed in 384-well microtiter plates by using gene-specific TaqMan probe and primer sets (Assays-on-Demand, Applied Biosystems, Foster City, CA) and an ABI PRISM 7900HT Sequence Detection System (Applied Biosystems). Template cDNA was added to the reaction mixture, and amplifications were initiated with a 10 min template denaturation step at 95°C, followed by 40 cycles at 95°C for 15 sec and 60°C for 1 min. All samples were amplified in triplicate. Data were analyzed with the Sequence Detector Software (Applied Biosystems). 

### 2.6. Immunoblotting

MSCs were washed with cold PBS and lysed in 300 *μ*L cold RIPA buffer (50 mM Tris-HCl, pH 7.5, containing 1% Triton X-100, 150 mM NaCl, 0.1% sodium dodecyl sulfate (SDS), 1% sodium deoxycholate, and a protease inhibitor cocktail (Thermo Fisher Scientific, Rockford, IL)). Cell lysates were centrifuged at 3,000 ×g for 15 min at 4°C. The supernatant was collected, and protein concentrations were analyzed using a bicinchoninic acid protein assay kit (Thermo Fisher Scientific). For electrophoresis, proteins (50 *μ*g) were dissolved in sample buffer (60 mM Tris-HCl, pH 6.8, containing 14.4 mM *β*-mercaptoethanol, 25% glycerol, 2% SDS, and 0.1% bromophenol blue), boiled for 5 min, and separated on a 10% SDS reducing gel. Separated proteins were transferred onto polyvinylidene difluoride membranes (GE Healthcare, Buckinghamshire, UK) using a trans-blot system (Invitrogen-Gibco). Blots were blocked for 1 h at RT in Tris-buffered saline (TBS) (10 mM Tris-HCl, pH 7.5, plus 150 mM NaCl) containing 5% nonfat dry milk (BD Biosciences), washed three times with TBS, and incubated at 4°C overnight with primary antibodies in TBST (TBS plus 0.01% Tween 20) containing 3% nonfat dry milk. On the next day, blots were washed three times with TBST and incubated for 1 h with secondary antibodies in TBST containing 3% nonfat dry milk at RT. After washing three times with TBST, proteins were visualized with an enhanced chemiluminescence detection system (GE Healthcare).

### 2.7. Immunocytochemistry

MSCs (200 and 5000 cells/cm^2^) were plated on individual coverslips and cultured for 7 d. On day 7, cells were placed into fixative solution (4% paraformaldehyde in PBS) for 30 min at RT in the dark and then washed three times with PBS. To detect internally expressed molecules, the cells were permeabilized with 0.25% Triton X-100 in PBS for 5 min at RT in the dark. Cells were washed three times and then incubated in blocking solution (5% FBS in PBS) for 1 h at RT. After another washing step, antibodies against PTGES, as well as ULBP1 (1 : 100), all purchased from Santa Cruz Biotechnology (Santa Cruz, CA), were added and incubated with the cells for 1 h at RT. The cells were washed three times and incubated with Alexa Fluor 488-conjugated secondary goat anti-mouse IgG (Invitrogen-Gibco) for 1 h at RT. Next, cells were washed three times and mounted in 4′,6-diamidino-2-phenylindole- (DAPI-) containing antifade mounting solution (Vectashield; Vector Lab., Burlingame, CA). Images of the cells were obtained using a Carl Zeiss LSM 700 confocal microscope system (Jena, Germany).

### 2.8. RNA Interference

MSCs were plated 24 h before small interfering RNA (siRNA) transfection so as to reach 50% confluence on the day of transfection. Cells were incubated with siRNA-lipofectamine 2000 (Invitrogen) complex for 18 h at 37°C. The medium was then changed, and the transfected cells were incubated for additional 12 h until the target gene was effectively downregulated. SiRNAs targeting *PTGES* (sc-430005) and *ULBP1* (sc-41642) and scrambled siRNA (sc-37007) were all purchased from Santa Cruz Biotechnology.

### 2.9. T-Cell Proliferation: BrdU Incorporation Assay and CFSE Staining

MSCs were seeded at 1.25 × 10^4^ cells/mL in 96-well plates in high glucose DMEM supplemented with 10% FBS. After 24 h, 10 *μ*g/mL Mitomycin-C (Sigma-Aldrich) was added to inhibit cell proliferation. The treated cells were incubated for additional 2 h at 37°C and then washed five times with culture media. Next, 1 × 10^5^ human peripheral blood mononuclear cells (hPBMCs) were isolated by gradient centrifugation, added to each well, and stimulated with 1 *μ*g/mL phytohemagglutinin (PHA; Sigma-Aldrich) to activate T-cell proliferation. The PHA-activated hPBMCs were then cultured on differentially conditioned MSCs for 3-4 days before the addition of 5-bromo-2-deoxyuridine (BrdU). Proliferation levels were assessed after 18 h using an assay kit from Roche Applied Science (Mannheim, Germany). For flow cytometry analysis of T-cell proliferation, hPBMCs were incubated with PBS containing 5 *μ*M carboxyfluorescein succinimidyl ester (CFSE, Invitrogen-Gibco) and 0.1% bovine serum albumin (Invitrogen-Gibco) for 10 min at 37°C. PHA-activated hPBMCs were then cultured on differentially conditioned MSCs for 3-4 days. Next, hPBMCs were incubated with a PE-conjugated anti-CD3 antibody (BD Biosciences) for 20 min at RT. The intensity of CFSE staining of PHA-induced proliferating CD3-positive T-cells was evaluated by flow cytometry (FACScan). Data were analyzed using CELLQUEST software.

### 2.10. Statistical Analysis

Data were expressed as the mean ± standard deviation (SD). The statistical significance of the microarray analysis results was determined as described in Section 2.4 earlier. The results were considered significant when *P* < 0.05.

## 3. Results

### 3.1. Characteristics of Isolated MSCs

**Figure 1:**
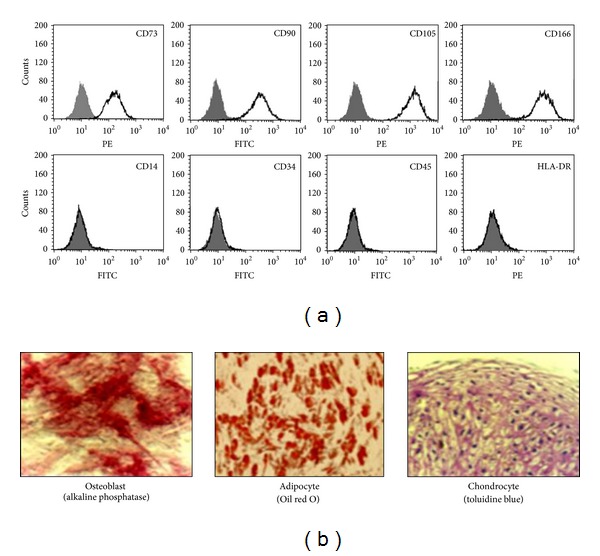
Characteristics of isolated MSCs. (a) Flow cytometric analysis of MSC surface markers. The expression of surface antigens was plotted against appropriate IgG isotype controls (grey histogram). MSCs used for the analyses were positive for CD73, CD90, CD105, and CD166 and negative for CD14, CD34, CD45, and HLA-DR (clear histogram). (b) Mesenchymal differentiation potential of MSCs. The cells derived from MSCs expressed alkaline phosphatase activity, stained positively for lipid vacuoles with Oil red O, and were positive for chondrocyte matrix with toluidine blue, indicating osteogenic, adipogenic, and chondrogenic differentiation, respectively.

**Figure 2:**
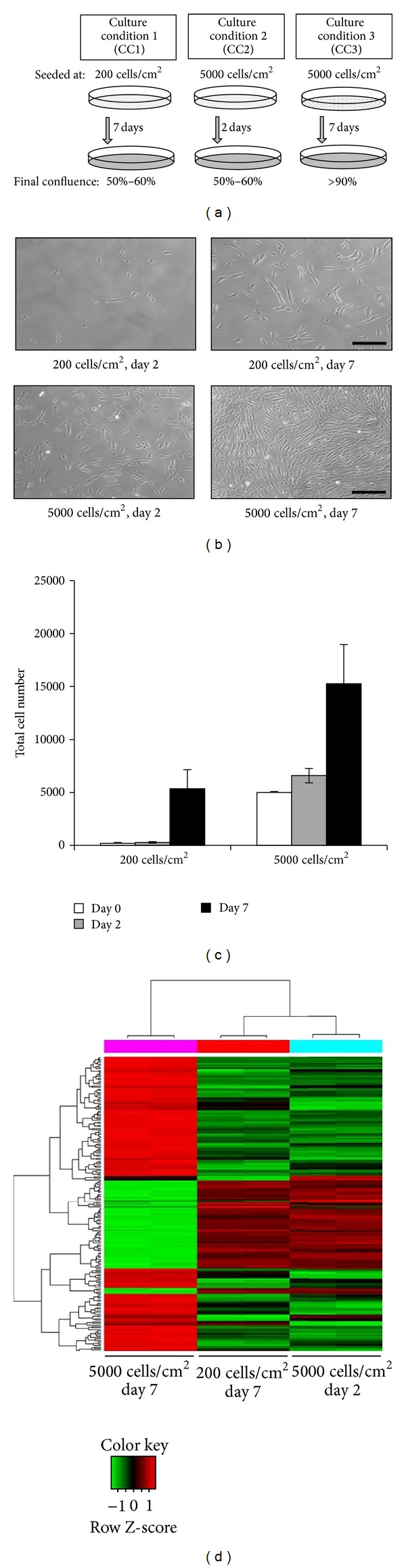
Characterization of MSCs cultured under different conditions. (a) Schematic diagram representing the three initial cell culture conditions. (b) Morphological appearance of MSCs under the three different culture conditions. Scale bar: 200 *μ*m. (c) Approximate number of MSCs cultured under the three different conditions at the time of harvest. (d) Hierarchical clustering analysis of differentially expressed genes. MSCs at passage 2 were used for microarray analysis. Each row represents a single gene, while each column represents the gene expression levels for each individual culture condition (*n* = 2 independent samples for each culture condition analyzed). The color corresponding to the level of gene expression varies from red for the lowest level of expression to green for the highest level of expression.

MSCs isolated from BM aspirates were characterized by flow cytometric analysis of expressed surface antigens; the cells were uniformly positive for CD73, CD90, CD105, and CD166 and negative for CD14, CD34, CD45, and HLA-DR (Figure 1(a)). Their differentiation potential was then assessed using osteogenic, adipogenic, and chondrogenic induction media; the cells differentiated into all three lineages (Figure 1(b)). MSCs were then plated in two different initial cell densities, which incubated for 2 d or 7 d, as shown in Figure 2(a): CC1, 200 cells/cm^2^ cultured for 7 d; CC2, 5,000 cells/cm^2^ cultured for 2 d; and CC3, 5,000 cells/cm^2^ cultured for 7 d. Elongated- and spindle-shaped cells were visible when CC1 and CC2 reached ~50% confluence. Cell morphology of MSCs in CC3 was similar to CC1 and CC2, but extensive cell-to-cell contacts were observed (Figure 2(b)). The approximate yields for cells plated at a density of 200 cells/cm^2^ or 5,000 cells/cm^2^ and cultured for various periods of time are shown in Figure 2(c).

### 3.2. Gene Expression Profiles of MSC Harvested at High *versus* Low Density

**Figure 3:**
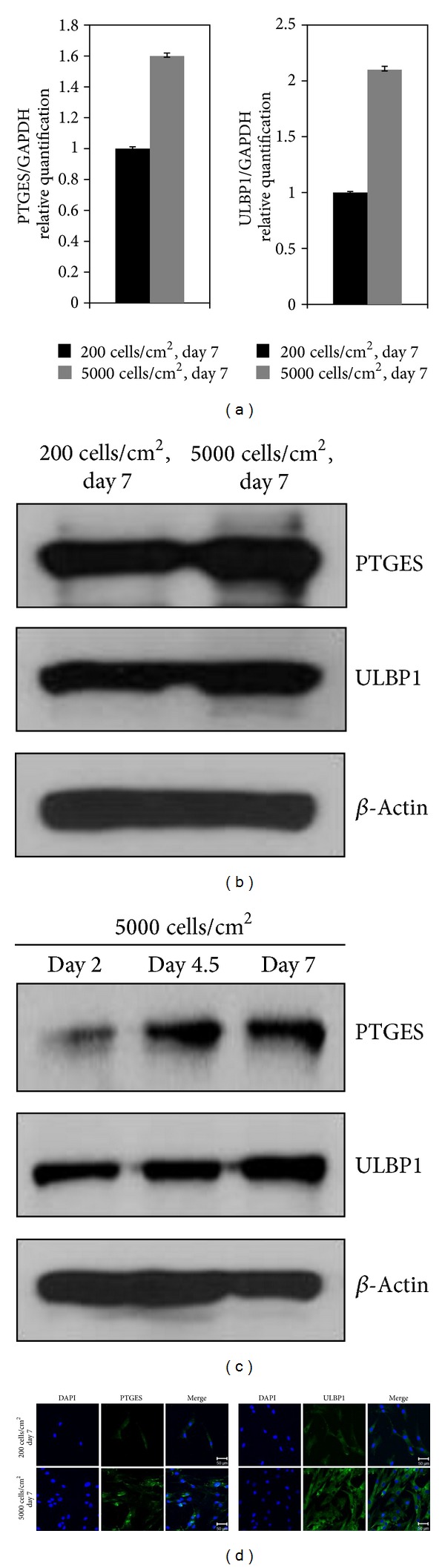
The expression levels of PTGES and ULBP1 in MSCs harvested at high cell density. (a) Quantitative RT-PCR analysis of selected genes upregulated in MSCs harvested at high cell density. (b) Immunoblot analysis of PTGES and ULBP1 protein expression in low and high density MSC cultures. *β*-Actin was used as a loading control. (c) Upregulated protein expression levels of PTGES and ULBP1 over time. (d) Immunocytochemistry showing higher expression of PTGES and ULBP1 in high *versus* low density MSCs. Scale bar: 50 *μ*m. MSCs at passage 3 or 4 were used to validate the expression levels of PTGES and ULBP1. Each experiment was independently performed using different MSC preparations.

Microarray analysis was performed to investigate whether *ex vivo* culture conditions affected MSC characteristics. Gene expression profiles varied depending on the level of cell confluence at harvest regardless of the initial seeding density. MSCs harvested at ~50% confluent CC1 and CC2 shared similar gene expression profiles, while MSCs of CC3 with ~90% confluence showed different gene expression profiles compared to CC1 and CC2 (*P* < 0.05, Figure 2(d)). These findings were consistent when the microarray analysis was repeated with MSCs isolated from a different donor (data not shown). MSCs of CC1 and CC3, which have been cultured for 7 days, were selected for comparative analyses of their gene expression profiles. Among the 24,526 genes analyzed, 276 genes were more highly expressed in MSCs harvested at ~90% (CC3 MSCs) versus ~50% confluence (CC1 MSCs), whereas 230 genes were down-regulated (*P* < 0.05, FC > 2, Table S1 in Supplementary Material available online at http://dx.doi.org/10.1155/2013/154919). Among the 276 genes upregulated in CC3 MSCs, PTGES and ULBP1 were selected for further study (Table 1); their changes in mRNA expression levels (confirmed by qRT-PCR) were 1.64- and 2.10-fold, respectively (Figure 3(a)). The protein expression levels of PTGES and ULBP1 were also investigated in CC1 and CC3 MSCs by western blotting. The proteins were all upregulated in MSCs harvested at ~90% compared with ~50% confluence (Figure 3(b)). Additionally, expression levels of PTGES and ULBP1 gradually increased with culture duration (Figure 3(c)). Furthermore, confocal microscopy confirmed that MSCs expressed higher levels of PTGES and ULBP1 when they were more confluent (Figure 3(d)). 

### 3.3. Mitogen-Induced T-Cell Proliferation in Response to Highly Confluent MSCs

**Figure 4:**
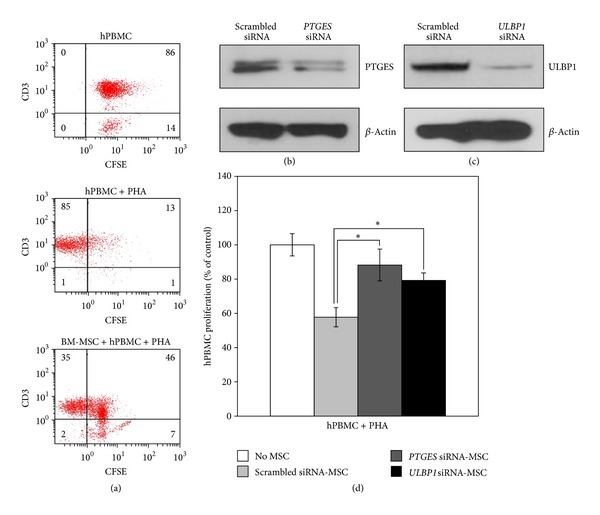
Immunosuppressive potential of MSCs harvested at high cell density. (a) The intensity of CFSE in PHA-induced proliferating CD3-positive T-cells was measured by flow cytometry. The proliferation of CD3-positive T-cell decreased in the coculture with high density MSCs. Immunoblot analysis of MSCs treated with *PTGES* (b) or *ULBP1* (c) siRNA. (d) The effect of high density MSCs transfected with *PTGES* or *ULBP1* siRNA on the response of T-cells to PHA was determined by the BrdU incorporation assay. MSCs at passage 3 or 4 were used to validate the correlation between the immunosuppressive effects of MSCs and the expression of specific genes. Each experiment was independently performed using different passage MSCs. Data are shown as the mean ± SD from three separate experiments. *P < 0.01.

To evaluate whether highly confluent MSCs affect the proliferation of T-cells *in vitro*, we measured the intensity of CFSE in mitogen-induced proliferating T-cells. When CFSE-labeled and PHA-activated PBMCs were incubated on a layer of highly confluent BM-MSCs, CD3-positive T-cell proliferation decreased significantly (Figure 4(a)). In addition, *PTGES *and *ULBP1* siRNAs were employed to interfere with the expression of each mRNA (Figures 4(b) and 4(c)) in order to evaluate the role of these proteins in immunosuppression of MSCs via a T-cell proliferation assay. We measured the incorporation of the thymidine analog BrdU in PHA-induced proliferating T-cells. When activated hPBMCs were incubated on a layer of MSCs harvested at ~90% confluence, the proliferation of T-cells decreased significantly to 57.7%. However, when the expression levels of PTGES and ULBP1 were individually down-regulated by siRNA transfection, hPBMC proliferation was restored to 88.2% and 79.2%, respectively (Figure 4(d)). 

## 4. Discussion

Previous reports suggest that *ex vivo* expansion of MSCs may be critical for positive results in clinical trials. Among various growth conditions, cell confluence is suggested to be a primary factor affecting the characteristics of highly heterogonous MSCs [13, 14]. Low initial plating density is considered to be beneficial for *ex vivo* MSC expansion, but most studies have investigated the effect of initial plating density on the capability of MSCs to further differentiate or expand, but not on the effect on their biological functions *in vivo* [14–17].

This study compared the gene expression profiles of MSCs collected at high *versus* low cell confluences. The density at harvest time could affect the gene expression profile. However, these densities could vary depending both on initial plating density and culture time. Although the pattern of gene expression of CC1 (200 cells/cm^2^ and cultured for 7 days) and CC2 (5,000 cells/cm^2^ and cultured for 2 days) harvested at same density share common pattern of gene expression compared to CC3 (5,000 cells/cm^2^ and cultured for 7 days), they are not exactly the same (Figure 2(d)). The same pattern of gene expression would not be always expected when the cell densities at harvest are the same. Some genes might be affected by culture time and be expressed in different ways. Thus, the initial plating density and culture time are two important parameters that might affect gene expression profile of MSCs at harvest time. In this study, harvesting MSCs at high cell confluence (~90%) appears to be beneficial for the collection of MSCs with enhanced immunosuppressive properties. This notion may be explained by the fact that the cells harvested at lower density (~50%–60%, CC1) tend to express a large number of genes regulating cell proliferation compared with cells harvested at high density (CC3). In fact, 29 genes known to be associated with proliferation, differentiation, and cell cycling were upregulated in CC1 MSCs, while only four of these genes were upregulated in CC3 MSCs. By contrast, highly confluent MSCs were more likely to express genes that are involved in other types of functional processes, including immunosuppression, because they are not able to further proliferate after reaching a certain density. In general, the genes highly expressed in CC3 MSCs were involved in processes such as chemotaxis, inflammation, and immune responses, indicating direct or indirect involvement in MSC immunomodulatory functions. However, the genes highly expressed in CC1 MSCs largely corresponded to cell differentiation- and proliferation-associated genes. Thus, harvesting MSCs at a high cell density (>90%) may be beneficial to enhance their immunosuppressive properties.

Based on our result of microarray analyses and previous reports [18–23], in this study, several genes related to the immunosuppressive properties of MSCs were selected, and these included PTGES and ULBP. These genes were upregulated in highly confluent (>90%) MSCs in this study and are reported to directly and/or indirectly affect the immunomodulatory activities of MSCs. Arachidonic acid (AA) is converted to prostaglandin (PG) H_2_, which is further converted to active PGE_2_ by PTGES. PEG_2_, a small lipid molecule that regulates numerous process in the body, has an inhibitory role over the maturation of dendritic cells and a direct effect on the proliferation and cytokine production of T-cells, by inhibiting polyamine synthesis, intracellular calcium release, activity of protein tyrosine kinase p59 fyn, and secretion of interleukin- (IL-) 2 [18–20]. The secreted PEG_2_ from MSCs also inhibits the secretion of proinflammatory cytokines and increases the secretion of anti-inflammatory cytokines by the stimulated macrophages, indicating that PEG_2_ directs stimulated macrophages into an anti-inflammatory phenotype [21]. In addition, ULBP1 is a ligand for the natural-killer group 2, member D (NKG2D) receptor, and the interaction of ULBP1 and NKG2D is reportedly essential for the delivery of activating signals to NK cells and/or the regulation of T-cell-receptor- (TCR-) mediated activation of T-cells [22, 23]. Furthermore, various cytokines (e.g., IL-15 and IL-17) are involved in signaling pathways in which ULBP1 takes part, although definite mechanisms and signals regulating their activation remain to be elucidated. We previously reported that activated T-cells express high levels of interferon- (IFN-) *γ* and that this level is significantly reduced when these cells are cocultured with MSCs [24]. Cytokines play a crucial role in regulating MSC-mediated immunosuppression. Zappia and colleagues showed that MSCs can reduce the serum levels of INF-*γ* and TNF-*α* produced by activated T-cells [25]. IFN-*γ* is an important cytokine that regulates the immunomodulatory functions of MSCs. Compelling studies have shown that IFN-*γ* plays an active role in MSC-mediated immunosuppression [26, 27]. In addition, treatment of MSCs with IFN-*γ* affects the levels of hepatocyte growth factor (HGF), IL-10, TGF-*β*_1_, cyclooxygenase- (COX-) 1, and COX-2 expression. We observed that T-cell proliferation was partially inhibited by MSCs in trans-well cultures, indicating that soluble factors, as well as cell-cell contacts, are involved in mediating the immunosuppressive properties of MSCs. Contact-dependent mechanisms, including MSC expression of B7H1, have been implicated in the immunosuppressive properties of MSCs [28, 29]. Soluble factors secreted by MSCs or by immune cells in response to MSCs play major roles in MSC-mediated immune suppression. Soluble factors such as HGF, PGE_2_, TGF-*β*_1_, indoleamine 2,3-dioxygenase (IDO), nitric oxide, and IL-10 have been implicated in this process, while other factors remain to be identified [30–34]. In the current study, siRNA experiments clearly illustrated the inhibitory roles of ULBP1 and PTGES in the regulation of T-cell proliferation. Thus, ULBP1 may play a role in cell-cell contacts and PTGES may be involved in the secretion of a soluble factor, such as PGE_2_, to mediate the immunosuppressive functions of MSCs.

The well-known IFN-*γ*-induced gene IDO is markedly upregulated in IFN-*γ*-preconditioned MSCs, and IDO suppresses antigen-driven proliferation of T-cells [35, 36]. We confirmed that IFN-*γ* stimulates the expression of IDO in MSCs derived from various sources in addition to BM, including human umbilical cord blood, adipose tissue, and Wharton's jelly [24]. The immunosuppressive activities of IDO are mediated by degradation of tryptophan, an amino acid that is essential for T-cell proliferation [37]. Therefore, priming MSCs with IFN-*γ*, in addition to harvesting highly confluent MSCs, is expected to enhance the immunomodulatory properties of these cells.

In conclusion, our findings are expected to provide useful guidelines for the collection of functionally qualified MSCs that can be more readily adapted for further uses, including therapies for graft versus host disease.

## Supplementary Material

Supplementary Table: List of genes that were differentially expressed in MSCs harvested at high cell confluence and at low cell confluence. Among the 24,526 genes analyzed, 276 genes were more highly expressed in MSCs harvested at *∼*90% (CC3 MSCs) versus *∼*50% confluence (CC1 MSCs), whereas 230 genes were down-regulated (*P* < 0.05, Fold Change > 2).

## Figures and Tables

**Table 1:** List of selected genes highly expressed in MSCs harvested at high cell confluence (>90%).

Abbr.*	Full name	FC	Adjusted *P*-value	Biological function	Gene ontology category
PTGES	Prostaglandin E synthase	2.40	<1.00*E* − 04	Involved in TP53-induced apoptosis and acute inflammatory responses [16]	GO:0006693_prostaglandin metabolic process GO:0007165_signal transduction

ULBP1	UL16 binding protein	2.01	<1.00*E* − 04	Associated with NKG2D-mediated immunity [18]	GO:0019882_antigen processing and presentation GO:0050776_regulation of immune response

*Abbr.: abbreviation.

Fold change (FC) is for comparison between MSCs harvested at high cell confluence (~90%) and at low cell confluence (~50%). Positive values indicate higher expression in MSCs harvested at high cell density.
