# Supplementary material for: Effect of Ex Vivo Culture Conditions on Immunosuppression by Human Mesenchymal Stem Cells
Source: Biomed Res Int. 2013 Jun 4;2013:154919. doi: 10.1155/2013/154919 (PMC3687591; doi:10.1155/2013/154919)
Supplement: Supplementary file 1 — Supplementary Table: List of genes that were differentially expressed in MSCs harvested at high cell confluence and at low cell confluence. Among the 24,526 genes analyzed, 276 genes were more highly expressed in MSCs harvested at ∼90% (CC3 MSCs) versus ∼50% confluence (CC1 MSCs), whereas 230 genes were down-regulated (P < 0.05, Fold Change > 2). [file 154919.f1.pdf]

Table S1. List of genes that were differentially expressed in MSCs harvested at high cell confluence (~90%, CC3) and at low cell confluence (~50%, CC1).

Gene Symbol	Gene Description	Fold Change
**PTGDS**	prostaglandin D2 synthase 21kDa (brain)	**13.94**
**PALM**	paralemmin	**12.01**
**CXCR7**	chemokine (C-X-C motif) receptor 7	**10.81**
**LEPR**	leptin receptor	**10.53**
**PTGIS**	prostaglandin I2 (prostacyclin) synthase	**10.49**
**COL11A1**	collagen, type XI, alpha 1	**10.47**
**NDUFA4L2**	NADH dehydrogenase (ubiquinone) 1 alpha subcomplex, 4-like 2	**10.22**
**VCAM1**	vascular cell adhesion molecule 1	**10.19**
**COMP**	cartilage oligomeric matrix protein	**10.10**
**MXRA5**	matrix-remodelling associated 5	**9.86**
**ECM2**	extracellular matrix protein 2, female organ and adipocyte specific	**9.84**
**FNDC1**	fibronectin type III domain containing 1	**8.87**
**C1R**	complement component 1, r subcomponent	**8.70**
**WISP2**	WNT1 inducible signaling pathway protein 2	**8.64**
**SERPINA3**	serpin peptidase inhibitor, clade A (alpha-1 antiproteinase, antitrypsin), member 3	**8.62**
**APOE**	apolipoprotein E	**8.20**
**CFD**	complement factor D (adipsin)	**7.60**
**C1S**	complement component 1, s subcomponent	**7.49**
**MFAP4**	microfibrillar-associated protein 4	**6.69**
**GDF15**	growth differentiation factor 15	**6.69**
**COL8A2**	collagen, type VIII, alpha 2	**6.68**
**EGR2**	early growth response 2 (Krox-20 homolog, Drosophila)	**6.40**
**DHRS3**	dehydrogenase/reductase (SDR family) member 3	**6.26**
**KRT17**	keratin 17	**5.98**
**SERPING1**	serpin peptidase inhibitor, clade G (C1 inhibitor), member 1	**5.77**
**DEPDC6**	DEP domain containing 6 (DEPDC6)	**5.75**
**ABCA1**	ATP-binding cassette, sub-family A (ABC1), member 1	**5.49**
**MGP**	matrix Gla protein	**5.35**
**MKX**	mohawk homeobox	**5.19**
**TRIB3**	tribbles homolog 3 (Drosophila)	**5.18**
**MME**	membrane metallo-endopeptidase	**5.09**
**PODN**	podocan	**5.06**
**MSC**	musculin (activated B-cell factor-1)	**5.04**
**FBXO32**	F-box protein 32	**4.89**
**IFITM1**	interferon induced transmembrane protein 1 (9-27)	**4.78**
**CYGB**	cytoglobin	**4.73**
**C5orf46**	chromosome 5 open reading frame 46	**4.72**
**THBS2**	thrombospondin 2	**4.67**
**FAM129A**	family with sequence similarity 129, member A	**4.57**
**ANKRD37**	ankyrin repeat domain 37	**4.56**
**CDKN2B**	cyclin-dependent kinase inhibitor 2B (p15, inhibits CDK4)	**4.55**
**PLXDC2**	plexin domain containing 2	**4.47**

Table S1. Continued

Gene Symbol	Gene Description	Fold Change
**TNS3**	tensin 3	**4.31**
**EFEMP1**	EGF-containing fibulin-like extracellular matrix protein 1	**4.30**
**DDIT4**	DNA-damage-inducible transcript 4	**4.24**
**COL12A1**	collagen, type XII, alpha 1	**4.20**
**FRAS1**	Fraser syndrome 1	**4.20**
**MMP11**	matrix metallopeptidase 11 (stromelysin 3)	**4.08**
**CPZ**	carboxypeptidase Z	**4.07**
**ADM2**	adrenomedullin 2	**4.01**
**AKR1C3**	aldo-keto reductase family 1, member C3 (3-alpha hydroxysteroid dehydrogenase, type II)	**3.97**
**CALB2**	calbindin 2	**3.94**
**CNTNAP2**	contactin associated protein-like 2	**3.90**
**SCRG1**	scrapie responsive protein 1	**3.89**
**TIMP3**	TIMP metallopeptidase inhibitor 3	**3.88**
**DMKN**	dermokine	**3.75**
**DCN**	decorin	**3.72**
**C10orf10**	chromosome 10 open reading frame 10	**3.66**
**FBLN2**	fibulin 2	**3.66**
**ITGA11**	integrin, alpha 11	**3.62**
**CTSF**	cathepsin F	**3.62**
**CCPG1**	cell cycle progression 1	**3.61**
**P8**	p8 protein (candidate of metastasis 1)	**3.58**
**SLC6A9**	solute carrier family 6 (neurotransmitter transporter, glycine), member 9	**3.55**
**MN1**	meningioma (disrupted in balanced translocation) 1	**3.53**
**IGFBP2**	insulin-like growth factor binding protein 2, 36kDa	**3.52**
**UNC5B**	unc-5 homolog B (C. elegans)	**3.51**
**KRT16**	keratin 16 (focal non-epidermolytic palmoplantar keratoderma)	**3.47**
**CTSK**	cathepsin K	**3.47**
**FLG**	filaggrin	**3.47**
**POSTN**	periostin, osteoblast specific factor	**3.43**
**RBP1**	retinol binding protein 1, cellular	**3.42**
**NUPR1**	nuclear protein 1	**3.41**
**SETBP1**	SET binding protein 1	**3.39**
**XAF1**	XIAP associated factor 1	**3.32**
**VLDLR**	very low density lipoprotein receptor	**3.32**
**ANKRD33**	ankyrin repeat domain 33	**3.30**
**DACT1**	dapper, antagonist of beta-catenin, homolog 1 (Xenopus laevis)	**3.30**
**PPP1R14C**	protein phosphatase 1, regulatory (inhibitor) subunit 14C	**3.28**
**THRA**	thyroid hormone receptor, alpha (erythroblastic leukemia viral (v-erb-a) oncogene homolog, avian)	**3.28**
**C1QTNF1**	C1q and tumor necrosis factor related protein 1	**3.24**
**FTHL12**	ferritin, heavy polypeptide-like 12	**3.19**
**PAPPA**	pregnancy-associated plasma protein A, pappalysin 1	**3.18**
**TPD52L1**	tumor protein D52-like 1	**3.17**

Table S1. Continued

Gene Symbol	Gene Description	Fold Change
**SULF1**	sulfatase 1	**3.16**
**PI16**	peptidase inhibitor 16	**3.15**
**C10orf54**	chromosome 10 open reading frame 54	**3.13**
**ALDOC**	aldolase C, fructose-bisphosphate	**3.13**
**SBSN**	suprabasin	**3.13**
**THBS3**	thrombospondin 3	**3.13**
**PSAT1**	phosphoserine aminotransferase 1	**3.09**
**MUC1**	mucin 1, cell surface associated	**3.09**
**COL3A1**	collagen, type III, alpha 1	**3.08**
**PMEPA1**	prostate transmembrane protein, androgen induced 1	**3.06**
**SORBS2**	sorbin and SH3 domain containing 2	**3.04**
**SLIT3**	slit homolog 3 (Drosophila)	**3.03**
**ASS1**	argininosuccinate synthetase 1	**2.95**
**NDRG1**	N-myc downstream regulated gene 1	**2.95**
**TMEM119**	transmembrane protein 119	**2.95**
**COL18A1**	collagen, type XVIII, alpha 1	**2.95**
**CRABP2**	cellular retinoic acid binding protein 2	**2.93**
**SMOC1**	SPARC related modular calcium binding 1	**2.92**
**PPP1R3C**	protein phosphatase 1, regulatory (inhibitor) subunit 3C	**2.92**
**MTHFD2**	methylenetetrahydrofolate dehydrogenase (NADP+ dependent) 2, methenyltetrahydrofolate cyclohydrolase	**2.91**
**FBLN1**	fibulin 1	**2.89**
**PCK2**	phosphoenolpyruvate carboxykinase 2 (mitochondrial)	**2.87**
**SCG2**	secretogranin II (chromogranin C)	**2.85**
**PIK3IP1**	phosphoinositide-3-kinase interacting protein 1	**2.84**
**DDIT4L**	DNA-damage-inducible transcript 4-like	**2.84**
**MAN1C1**	mannosidase, alpha, class 1C, member 1	**2.84**
**PROS1**	protein S (alpha)	**2.82**
**MX1**	myxovirus (influenza virus) resistance 1, interferon-inducible protein p78 (mouse)	**2.82**
**GPNMB**	glycoprotein (transmembrane) nmb	**2.81**
**TXNIP**	thioredoxin interacting protein	**2.80**
**OLFML1**	olfactomedin-like 1	**2.78**
**FMOD**	fibromodulin	**2.77**
**CBS**	cystathionine-beta-synthase	**2.77**
**ZSWIM4**	zinc finger, SWIM-type containing 4	**2.75**
**SESN2**	sestrin 2	**2.74**
**AGT**	angiotensinogen (serpin peptidase inhibitor, clade A, member 8)	**2.74**
**ASNS**	asparagine synthetase	**2.69**
**NNMT**	nicotinamide N-methyltransferase	**2.67**
**PLAC9**	placenta-specific 9	**2.67**
**CEBPD**	CCAAT/enhancer binding protein (C/EBP), delta	**2.66**
**SOX4**	SRY (sex determining region Y)-box 4	**2.65**
**HEPH**	hephaestin	**2.62**

Table S1. Continued

Gene Symbol	Gene Description	Fold Change
**LAMA5**	laminin, alpha 5	**2.62**
**FTHL3P**	ferritin, heavy polypeptide-like 3 pseudogene	**2.62**
**ABLIM1**	actin binding LIM protein 1	**2.62**
**KAL1**	Kallmann syndrome 1 sequence	**2.61**
**PRSS23**	protease, serine, 23	**2.60**
**HNMT**	histamine N-methyltransferase	**2.59**
**OSBPL10**	oxysterol binding protein-like 10	**2.58**
**TUBB2B**	tubulin, beta 2B	**2.57**
**CTSD**	cathepsin D	**2.57**
**RAB33A**	RAB33A, member RAS oncogene family	**2.56**
**CCL2**	chemokine (C-C motif) ligand 2	**2.54**
**CFB**	complement factor B	**2.53**
**GPC6**	glypican 6	**2.53**
**HLA-DMA**	major histocompatibility complex, class II, DM alpha	**2.53**
**DBC1**	deleted in bladder cancer 1	**2.52**
**SORCS2**	sortilin-related VPS10 domain containing receptor 2	**2.52**
**RPS29**	ribosomal protein S29	**2.50**
**SEZ6L2**	seizure related 6 homolog (mouse)-like 2	**2.50**
**MKNK2**	MAP kinase interacting serine/threonine kinase 2	**2.49**
**SUSD2**	sushi domain containing 2	**2.49**
**ANGPTL2**	angiopoietin-like 2	**2.49**
**FOXO1**	forkhead box O1	**2.48**
**SERPINF1**	serpin peptidase inhibitor, clade F (alpha-2 antiplasmin, pigment epithelium derived factor), member 1	**2.48**
**CYP1B1**	cytochrome P450, family 1, subfamily B, polypeptide 1	**2.48**
**ANKRD38**	ankyrin repeat domain 38	**2.47**
**FCGRT**	Fc fragment of IgG, receptor, transporter, alpha	**2.47**
**C1QTNF5**	C1q and tumor necrosis factor related protein 5	**2.47**
**NUAK1**	NUAK family, SNF1-like kinase, 1	**2.46**
**JAM2**	junctional adhesion molecule 2	**2.46**
**PDE5A**	phosphodiesterase 5A, cGMP-specific	**2.45**
**FAM43A**	family with sequence similarity 43, member A	**2.44**
**MFAP2**	microfibrillar-associated protein 2	**2.44**
**C9orf150**	chromosome 9 open reading frame 150	**2.44**
**SPRY1**	sprouty homolog 1, antagonist of FGF signaling (Drosophila)	**2.43**
**KCNE4**	potassium voltage-gated channel, Isk-related family, member 4	**2.43**
**EPSTI1**	epithelial stromal interaction 1 (breast)	**2.43**
**ANTXR1**	anthrax toxin receptor 1	**2.42**
**FTHL11**	ferritin, heavy polypeptide-like 11 on chromosome 8	**2.40**
**PTGES**	prostaglandin E synthase	**2.40**
**GAA**	glucosidase, alpha; acid	**2.40**
**CYBRD1**	cytochrome b reductase 1	**2.40**
**SVEP1**	sushi, von Willebrand factor type A, EGF and pentraxin domain containing 1	**2.40**

Table S1. Continued

Gene Symbol	Gene Description	Fold Change
**RARRES3**	retinoic acid receptor responder (tazarotene induced) 3	**2.39**
**PCMTD1**	protein-L-isoaspartate (D-aspartate) O-methyltransferase domain containing 1	**2.39**
**FAM38B**	family with sequence similarity 38, member B	**2.39**
**MAP3K8**	mitogen-activated protein kinase kinase kinase 8	**2.39**
**LTBP3**	latent transforming growth factor beta binding protein 3	**2.38**
**RRAGD**	Ras-related GTP binding D	**2.38**
**PLSCR4**	phospholipid scramblase 4	**2.36**
**GALNAC4S-6ST**	B cell RAG associated protein	**2.36**
**SLC38A1**	solute carrier family 38, member 1	**2.36**
**OAS2**	2'-5'-oligoadenylate synthetase 2, 69/71kDa	**2.35**
**GABARAPL1**	GABA(A) receptor-associated protein like 1	**2.35**
**ACAP1**	ArfGAP with coiled-coil, ankyrin repeat and PH domains 1	**2.35**
**CRLF1**	cytokine receptor-like factor 1	**2.34**
**MYO1D**	myosin ID	**2.34**
**FAM102A**	family with sequence similarity 102, member A	**2.33**
**PLEKHF1**	pleckstrin homology domain containing, family F (with FYVE domain) member 1	**2.32**
**P4HA1**	procollagen-proline, 2-oxoglutarate 4-dioxygenase (proline 4-hydroxylase), alpha polypeptide I	**2.32**
**CDH13**	cadherin 13, H-cadherin (heart)	**2.32**
**NCOA7**	nuclear receptor coactivator 7	**2.31**
**YPEL3**	yippee-like 3 (Drosophila)	**2.31**
**PPFIBP2**	PTPRF interacting protein, binding protein 2 (liprin beta 2)	**2.30**
**DPYSL4**	dihydropyrimidinase-like 4	**2.30**
**EPAS1**	endothelial PAS domain protein 1	**2.30**
**GPT2**	glutamic pyruvate transaminase (alanine aminotransferase) 2	**2.30**
**VCAN**	versican	**2.29**
**LBH**	limb bud and heart development homolog (mouse)	**2.28**
**TCEA2**	transcription elongation factor A (SII), 2	**2.27**
**COL16A1**	collagen, type XVI, alpha 1	**2.27**
**FAM116B**	family with sequence similarity 116, member B	**2.27**
**TMEM140**	transmembrane protein 140	**2.27**
**AK3L1**	adenylate kinase 3-like 1	**2.27**
**TCTN1**	tectonic family member 1	**2.26**
**BEXL1**	brain expressed X-linked-like 1	**2.25**
**ACVR2A**	activin A receptor, type IIA	**2.25**
**ANKRD29**	ankyrin repeat domain 29	**2.25**
**WARS**	tryptophanyl-tRNA synthetase	**2.24**
**FAM46A**	family with sequence similarity 46, member A	**2.24**
**SEMA3C**	sema domain, immunoglobulin domain (Ig), short basic domain, secreted, (semaphorin) 3C	**2.24**
**FLJ40504**	hypothetical protein FLJ40504	**2.23**
**HSD17B6**	hydroxysteroid (17-beta) dehydrogenase 6 homolog (mouse)	**2.23**
**RPS27**	ribosomal protein S27 (metallopanstimulin 1)	**2.22**
**CLDN1**	claudin 1	**2.22**

Table S1. Continued

Gene Symbol	Gene Description	Fold Change
**GBP2**	guanylate binding protein 2, interferon-inducible	**2.21**
**SLC22A15**	solute carrier family 22 (organic cation transporter), member 15	**2.21**
**BACE2**	beta-site APP-cleaving enzyme 2	**2.20**
**DRAM**	damage-regulated autophagy modulator	**2.20**
**ITGBL1**	integrin, beta-like 1 (with EGF-like repeat domains)	**2.20**
**SLC7A7**	solute carrier family 7 (cationic amino acid transporter, y+ system), member 7	**2.20**
**ADAMTSL4**	ADAMTS-like 4	**2.19**
**PGCP**	plasma glutamate carboxypeptidase	**2.19**
**FAP**	fibroblast activation protein, alpha	**2.18**
**PDGFRB**	platelet-derived growth factor receptor, beta polypeptide	**2.18**
**C5orf41**	chromosome 5 open reading frame 41	**2.18**
**RENBP**	renin binding protein	**2.17**
**HLA-F**	major histocompatibility complex, class I, F	**2.17**
**SSPN**	sarcospan (Kras oncogene-associated gene)	**2.17**
**SPATA18**	spermatogenesis associated 18 homolog (rat)	**2.17**
**TMTC1**	transmembrane and tetratricopeptide repeat containing 1	**2.17**
**GPRC5C**	G protein-coupled receptor, family C, group 5, member C	**2.16**
**CXCL16**	chemokine (C-X-C motif) ligand 16	**2.15**
**GATS**	opposite strand transcription unit to STAG3	**2.14**
**FAM110B**	family with sequence similarity 110, member B	**2.14**
**GAS1**	growth arrest-specific 1	**2.14**
**AZI2**	5-azacytidine induced 2	**2.14**
**LARP6**	La ribonucleoprotein domain family, member 6	**2.13**
**C7orf41**	chromosome 7 open reading frame 41	**2.13**
**BNIP3**	BCL2/adenovirus E1B 19kDa interacting protein 3	**2.13**
**SOX18**	SRY (sex determining region Y)-box 18	**2.12**
**INSIG2**	insulin induced gene 2	**2.12**
**KLF9**	Kruppel-like factor 9	**2.12**
**SPON2**	spondin 2, extracellular matrix protein	**2.12**
**ENPP2**	ectonucleotide pyrophosphatase/phosphodiesterase 2	**2.12**
**SPAG4**	sperm associated antigen 4	**2.12**
**RAB11FIP1**	RAB11 family interacting protein 1 (class I)	**2.11**
**VEGFA**	vascular endothelial growth factor A	**2.11**
**HBP1**	HMG-box transcription factor 1	**2.10**
**LHPP**	phospholysine phosphohistidine inorganic pyrophosphate phosphatase	**2.10**
**C19orf4**	chromosome 19 open reading frame 4	**2.09**
**FLYWCH1**	FLYWCH-type zinc finger 1	**2.09**
**GRN**	granulin	**2.08**
**DCLK1**	doublecortin-like kinase 1	**2.08**
**SLC25A23**	solute carrier family 25 (mitochondrial carrier; phosphate carrier), member 23	**2.07**
**IRF1**	interferon regulatory factor 1	**2.07**
**PLA2G4C**	phospholipase A2, group IVC (cytosolic, calcium-independent)	**2.07**
**ULK1**	unc-51-like kinase 1 (C. elegans)	**2.07**

Table S1. Continued

Gene Symbol	Gene Description	Fold Change
**ABHD4**	abhydrolase domain containing 4	**2.06**
**DDIT3**	DNA-damage-inducible transcript 3	**2.06**
**TMEM47**	transmembrane protein 47	**2.06**
**FBLN5**	fibulin 5	**2.06**
**SRPK2**	SFRS protein kinase 2	**2.06**
**C20orf39**	chromosome 20 open reading frame 39	**2.06**
**DNAJC12**	DnaJ (Hsp40) homolog, subfamily C, member 12	**2.05**
**OPLAH**	5-oxoprolinase (ATP-hydrolysing)	**2.05**
**MFAP5**	microfibrillar associated protein 5	**2.04**
**IFI27**	interferon, alpha-inducible protein 27	**2.04**
**CD82**	CD82 molecule	**2.04**
**CRBN**	cereblon	**2.04**
**DPYSL3**	dihydropyrimidinase-like 3	**2.03**
**VDR**	vitamin D (1,25- dihydroxyvitamin D3) receptor	**2.03**
**NME5**	non-metastatic cells 5, protein expressed in (nucleoside-diphosphate kinase)	**2.03**
**CPXM2**	carboxypeptidase X (M14 family), member 2	**2.02**
**PLD3**	phospholipase D family, member 3	**2.02**
**LY96**	lymphocyte antigen 96	**2.01**
**BNIP3L**	BCL2/adenovirus E1B 19kDa interacting protein 3-like	**2.01**
**JUNB**	jun B proto-oncogene	**2.01**
**ULBP1**	UL16 binding protein 1	**2.01**
**MXD1**	MAX dimerization protein 1	**2.01**
**CFH**	complement factor H	**2.00**
**GMPPB**	GDP-mannose pyrophosphorylase B	**-2.00**
**ITIH3**	inter-alpha (globulin) inhibitor H3	**-2.00**
**NRIP3**	nuclear receptor interacting protein 3	**-2.00**
**RFC4**	replication factor C (activator 1) 4, 37kDa	**-2.01**
**C14orf151**	chromosome 14 open reading frame 151	**-2.01**
**NEK2**	NIMA (never in mitosis gene a)-related kinase 2	**-2.01**
**PDCD1LG2**	programmed cell death 1 ligand 2	**-2.01**
**TRPV2**	transient receptor potential cation channel, subfamily V, member 2	**-2.01**
**WDR51A**	WD repeat domain 51A	**-2.01**
**BRCA1**	breast cancer 1, early onset	**-2.01**
**LMNB2**	lamin B2	**-2.01**
**PSG4**	pregnancy specific beta-1-glycoprotein 4	**-2.02**
**FMN2**	formin 2	**-2.02**
**RAD51**	RAD51 homolog (RecA homolog, E. coli) (S. cerevisiae)	**-2.03**
**WDR62**	WD repeat domain 62	**-2.03**
**LIN7A**	lin-7 homolog A (C. elegans)	**-2.03**
**HYI**	hydroxypyruvate isomerase homolog (E. coli)	**-2.03**
**RFC5**	replication factor C (activator 1) 5, 36.5kDa	**-2.04**
**GLIPR1**	GLI pathogenesis-related 1	**-2.04**
**EXOSC9**	exosome component 9	**-2.04**

Table S1. Continued

Gene Symbol	Gene Description	Fold Change
**CGB5**	chorionic gonadotropin, beta polypeptide 5	**-2.05**
**MND1**	meiotic nuclear divisions 1 homolog (S. cerevisiae)	**-2.05**
**NES**	nestin	**-2.05**
**TBX2**	T-box 2	**-2.05**
**DNAJC9**	DnaJ (Hsp40) homolog, subfamily C, member 9	**-2.05**
**RBL1**	retinoblastoma-like 1 (p107)	**-2.05**
**UNQ1940**	HWKM1940	**-2.05**
**POLE**	polymerase (DNA directed), epsilon	**-2.05**
**C13orf3**	chromosome 13 open reading frame 3	**-2.06**
**LBR**	lamin B receptor	**-2.06**
**C14orf80**	chromosome 14 open reading frame 80	**-2.06**
**E2F7**	E2F transcription factor 7	**-2.06**
**KIF15**	kinesin family member 15	**-2.06**
**FIGNL1**	fidgetin-like 1	**-2.06**
**XRCC3**	X-ray repair complementing defective repair in Chinese hamster cells 3	**-2.07**
**TTF2**	transcription termination factor, RNA polymerase II	**-2.08**
**NCAPD2**	non-SMC condensin I complex, subunit D2	**-2.08**
**DDX39**	DEAD (Asp-Glu-Ala-Asp) box polypeptide 39	**-2.09**
**TIMELESS**	timeless homolog (Drosophila)	**-2.09**
**DONSON**	downstream neighbor of SON	**-2.09**
**CENPN**	centromere protein N	**-2.09**
**IGF2BP3**	insulin-like growth factor 2 mRNA binding protein 3	**-2.09**
**C16orf59**	chromosome 16 open reading frame 59	**-2.10**
**HBEGF**	heparin-binding EGF-like growth factor	**-2.11**
**ATP2B4**	ATPase, Ca++ transporting, plasma membrane 4	**-2.11**
**CASC5**	cancer susceptibility candidate 5	**-2.11**
**TMPO**	thymopoietin	**-2.12**
**VEGFC**	vascular endothelial growth factor C	**-2.12**
**MPP4**	membrane protein, palmitoylated 4 (MAGUK p55 subfamily member 4)	**-2.13**
**CCDC99**	coiled-coil domain containing 99	**-2.13**
**DSN1**	DSN1, MIND kinetochore complex component, homolog (S. cerevisiae)	**-2.14**
**DNMT1**	DNA (cytosine-5-)-methyltransferase 1	**-2.14**
**FOSL1**	FOS-like antigen 1	**-2.15**
**LCP1**	lymphocyte cytosolic protein 1 (L-plastin)	**-2.15**
**INA**	internexin neuronal intermediate filament protein, alpha	**-2.16**
**TUBB2C**	tubulin, beta 2C	**-2.16**
**MYO19**	myosin XIX	**-2.16**
**FOXQ1**	forkhead box Q1	**-2.17**
**SPP1**	secreted phosphoprotein 1	**-2.17**
**CSTF3**	cleavage stimulation factor, 3' pre-RNA, subunit 3, 77kDa	**-2.18**
**DBNDD1**	dysbindin (dystrobrevin binding protein 1) domain containing 1	**-2.18**
**PENK**	proenkephalin	**-2.19**
**CDCA4**	cell division cycle associated 4	**-2.19**

Table S1. Continued

Gene Symbol	Gene Description	Fold Change
**PRIM1**	primase, DNA, polypeptide 1 (49kDa)	**-2.19**
**STX1A**	syntaxin 1A (brain)	**-2.19**
**KNTC1**	kinetochore associated 1	**-2.19**
**ITGA10**	integrin, alpha 10	**-2.19**
**FANCD2**	Fanconi anemia, complementation group D2	**-2.19**
**BTBD11**	BTB (POZ) domain containing 11	**-2.20**
**MCM10**	minichromosome maintenance complex component 10	**-2.21**
**POP1**	processing of precursor 1, ribonuclease P/MRP subunit (S. cerevisiae)	**-2.21**
**CCNB1**	cyclin B1	**-2.22**
**C3orf52**	chromosome 3 open reading frame 52	**-2.23**
**SMTN**	smoothelin	**-2.23**
**GABBR2**	gamma-aminobutyric acid (GABA) B receptor, 2	**-2.23**
**GSG2**	germ cell associated 2 (haspin)	**-2.23**
**ATAD3B**	ATPase family, AAA domain containing 3B	**-2.23**
**ATAD3A**	ATPase family, AAA domain containing 3A	**-2.24**
**HYLS1**	hydrolethalus syndrome 1	**-2.24**
**PPIL5**	peptidylprolyl isomerase (cyclophilin)-like 5	**-2.25**
**CHTF18**	CTF18, chromosome transmission fidelity factor 18 homolog (S. cerevisiae)	**-2.25**
**CENPL**	centromere protein L	**-2.25**
**C18orf24**	chromosome 18 open reading frame 24	**-2.26**
**C1orf112**	chromosome 1 open reading frame 112	**-2.27**
**EZH2**	enhancer of zeste homolog 2 (Drosophila)	**-2.27**
**CHAF1B**	chromatin assembly factor 1, subunit B (p60)	**-2.27**
**E2F2**	E2F transcription factor 2	**-2.28**
**CENPK**	centromere protein K	**-2.28**
**UHRF1**	ubiquitin-like with PHD and ring finger domains 1	**-2.29**
**LIG1**	ligase I, DNA, ATP-dependent	**-2.29**
**SMC4**	structural maintenance of chromosomes 4	**-2.30**
**KPNA2**	karyopherin alpha 2 (RAG cohort 1, importin alpha 1)	**-2.30**
**KIF14**	kinesin family member 14	**-2.31**
**POLA2**	polymerase (DNA directed), alpha 2 (70kD subunit)	**-2.31**
**UBE2T**	ubiquitin-conjugating enzyme E2T (putative)	**-2.32**
**HELLS**	helicase, lymphoid-specific	**-2.32**
**WNT7B**	wingless-type MMTV integration site family, member 7B	**-2.32**
**C17orf53**	chromosome 17 open reading frame 53	**-2.33**
**EDN1**	endothelin 1	**-2.33**
**CDCA1**	cell division cycle associated 1	**-2.36**
**MCM5**	minichromosome maintenance complex component 5	**-2.36**
**MKI67**	antigen identified by monoclonal antibody Ki-67	**-2.37**
**PLCXD1**	phosphatidylinositol-specific phospholipase C, X domain containing 1	**-2.37**
**ADRB2**	adrenergic, beta-2-, receptor, surface	**-2.39**
**RECQL4**	RecQ protein-like 4	**-2.39**
**POLE2**	polymerase (DNA directed), epsilon 2 (p59 subunit)	**-2.40**

Table S1. Continued

Gene Symbol	Gene Description	Fold Change
**BARD1**	BRCA1 associated RING domain 1	**-2.42**
**WWC1**	WW and C2 domain containing 1	**-2.43**
**SERPINB7**	serpin peptidase inhibitor, clade B (ovalbumin), member 7	**-2.43**
**RAB3B**	RAB3B, member RAS oncogene family	**-2.43**
**BLM**	Bloom syndrome	**-2.43**
**DIAPH3**	diaphanous homolog 3 (Drosophila)	**-2.44**
**CCNE2**	cyclin E2	**-2.45**
**CHEK1**	CHK1 checkpoint homolog (S. pombe)	**-2.45**
**SERPINB2**	serpin peptidase inhibitor, clade B (ovalbumin), member 2	**-2.45**
**MCM4**	minichromosome maintenance complex component 4	**-2.46**
**ORC1L**	origin recognition complex, subunit 1-like (yeast)	**-2.46**
**NUDT1**	nudix (nucleoside diphosphate linked moiety X)-type motif 1	**-2.46**
**FGF5**	fibroblast growth factor 5	**-2.47**
**CDC25C**	cell division cycle 25 homolog C (S. pombe)	**-2.48**
**RGMB**	RGM domain family, member B	**-2.48**
**INCENP**	inner centromere protein antigens 135/155kDa	**-2.48**
**ADARB1**	adenosine deaminase, RNA-specific, B1 (RED1 homolog rat)	**-2.52**
**FLJ35409**	PREDICTED: FLJ35409 protein	**-2.53**
**RFC3**	replication factor C (activator 1) 3, 38kDa	**-2.53**
**CDT1**	chromatin licensing and DNA replication factor 1	**-2.56**
**FOXE1**	forkhead box E1 (thyroid transcription factor 2)	**-2.56**
**CDC25A**	cell division cycle 25 homolog A (S. pombe)	**-2.56**
**CDK2**	cyclin-dependent kinase 2	**-2.57**
**IL11**	interleukin 11	**-2.57**
**EXO1**	exonuclease 1	**-2.58**
**KIF18A**	kinesin family member 18A	**-2.58**
**RAD51AP1**	RAD51 associated protein 1	**-2.58**
**KIF11**	kinesin family member 11	**-2.58**
**LYPD1**	LY6/PLAUR domain containing 1	**-2.58**
**PLK1**	polo-like kinase 1 (Drosophila)	**-2.59**
**MCM2**	minichromosome maintenance complex component 2	**-2.59**
**CENPE**	centromere protein E, 312kDa	**-2.59**
**SUV39H1**	suppressor of variegation 3-9 homolog 1 (Drosophila)	**-2.62**
**GPSM2**	G-protein signalling modulator 2 (AGS3-like, C. elegans)	**-2.63**
**PSMC3IP**	PSMC3 interacting protein	**-2.64**
**OIP5**	Opa interacting protein 5	**-2.64**
**ERCC6L**	excision repair cross-complementing rodent repair deficiency, complementation group 6-like	**-2.65**
**STIL**	SCL/TAL1 interrupting locus	**-2.65**
**H2AFX**	H2A histone family, member X	**-2.66**
**GPR56**	G protein-coupled receptor 56	**-2.66**
**PHF19**	PHD finger protein 19	**-2.67**
**MELK**	maternal embryonic leucine zipper kinase	**-2.67**

Table S1. Continued

Gene Symbol	Gene Description	Fold Change
**ASF1B**	ASF1 anti-silencing function 1 homolog B (S. cerevisiae)	**-2.67**
**KIAA0101**	KIAA0101	**-2.69**
**SPC24**	SPC24, NDC80 kinetochore complex component, homolog (S. cerevisiae)	**-2.71**
**GINS2**	GINS complex subunit 2 (Psf2 homolog)	**-2.71**
**RACGAP1**	Rac GTPase activating protein 1	**-2.73**
**HAS2**	hyaluronan synthase 2	**-2.73**
**PTTG3**	pituitary tumor-transforming 3	**-2.76**
**FEN1**	flap structure-specific endonuclease 1	**-2.77**
**CDC45L**	CDC45 cell division cycle 45-like (S. cerevisiae)	**-2.78**
**PBK**	PDZ binding kinase	**-2.80**
**NCAPG2**	non-SMC condensin II complex, subunit G2	**-2.80**
**CDCA7**	cell division cycle associated 7	**-2.81**
**CDCA3**	cell division cycle associated 3	**-2.84**
**TYMS**	thymidylate synthetase	**-2.86**
**KIF20B**	kinesin family member 20B	**-2.87**
**ANLN**	anillin, actin binding protein	**-2.90**
**CDKN3**	cyclin-dependent kinase inhibitor 3 (CDK2-associated dual specificity phosphatase)	**-2.91**
**PSRC1**	proline/serine-rich coiled-coil 1	**-2.91**
**CDC2**	cell division cycle 2, G1 to S and G2 to M	**-2.91**
**ASPM**	asp (abnormal spindle) homolog, microcephaly associated (Drosophila)	**-2.92**
**CCNF**	cyclin F	**-2.94**
**C11orf82**	chromosome 11 open reading frame 82	**-2.95**
**TACC3**	transforming, acidic coiled-coil containing protein 3	**-2.95**
**STMN1**	stathmin 1/oncoprotein 18	**-2.97**
**WNK4**	WNK lysine deficient protein kinase 4	**-2.98**
**KIF23**	kinesin family member 23	**-3.01**
**CENPM**	centromere protein M	**-3.02**
**CENPF**	centromere protein F, 350/400ka (mitosin)	**-3.03**
**MCM7**	minichromosome maintenance complex component 7	**-3.05**
**POLQ**	polymerase (DNA directed), theta	**-3.06**
**TOP2A**	topoisomerase (DNA) II alpha 170kDa	**-3.06**
**ATAD2**	ATPase family, AAA domain containing 2	**-3.09**
**SCARA3**	scavenger receptor class A, member 3	**-3.09**
**PTTG1**	pituitary tumor-transforming 1	**-3.11**
**BUB1**	BUB1 budding uninhibited by benzimidazoles 1 homolog (yeast)	**-3.11**
**C9orf140**	chromosome 9 open reading frame 140	**-3.14**
**NUSAP1**	nucleolar and spindle associated protein 1	**-3.15**
**PRR11**	proline rich 11	**-3.18**
**CCNB2**	cyclin B2	**-3.18**
**TTK**	TTK protein kinase	**-3.19**
**SGOL1**	shugoshin-like 1 (S. pombe)	**-3.19**
**TMSL8**	thymosin-like 8	**-3.21**
**APOBEC3B**	apolipoprotein B mRNA editing enzyme, catalytic polypeptide-like 3B	**-3.21**

Table S1. Continued

Gene Symbol	Gene Description	Fold Change
**KIF4A**	kinesin family member 4A	**-3.22**
**NCAPG**	non-SMC condensin I complex, subunit G	**-3.24**
**BIRC5**	baculoviral IAP repeat-containing 5	**-3.25**
**CTSC**	cathepsin C	**-3.27**
**FAM64A**	family with sequence similarity 64, member A	**-3.28**
**PKMYT1**	protein kinase, membrane associated tyrosine/threonine 1	**-3.28**
**TRIP13**	thyroid hormone receptor interactor 13	**-3.29**
**CENPA**	centromere protein A	**-3.31**
**PLK4**	polo-like kinase 4 (Drosophila)	**-3.36**
**CDCA5**	cell division cycle associated 5	**-3.38**
**PRC1**	protein regulator of cytokinesis 1	**-3.39**
**CDCA2**	cell division cycle associated 2	**-3.42**
**CEP55**	centrosomal protein 55kDa	**-3.45**
**TPX2**	TPX2, microtubule-associated, homolog (Xenopus laevis)	**-3.48**
**C11orf87**	chromosome 11 open reading frame 87	**-3.52**
**TK1**	thymidine kinase 1, soluble	**-3.53**
**GTSE1**	G-2 and S-phase expressed 1	**-3.53**
**DLG7**	discs, large homolog 7 (Drosophila)	**-3.56**
**HJURP**	Holliday junction recognition protein	**-3.57**
**KIF2C**	kinesin family member 2C	**-3.61**
**UBE2C**	ubiquitin-conjugating enzyme E2C	**-3.61**
**SHISA2**	shisa homolog 2 (Xenopus laevis)	**-3.63**
**PODXL**	podocalyxin-like	**-3.68**
**TROAP**	trophinin associated protein (tastin)	**-3.70**
**LMNB1**	lamin B1	**-3.75**
**CKAP2L**	cytoskeleton associated protein 2-like	**-3.76**
**FOXM1**	forkhead box M1	**-3.76**
**AURKB**	aurora kinase B	**-3.81**
**RRM2**	ribonucleotide reductase M2 polypeptide	**-4.05**
**AURKA**	aurora kinase A	**-4.05**
**KIF20A**	kinesin family member 20A	**-4.14**
**CDCA8**	cell division cycle associated 8	**-4.18**
**HMMR**	hyaluronan-mediated motility receptor (RHAMM)	**-4.23**
**KIFC1**	kinesin family member C1	**-4.24**
**CCNA2**	cyclin A2	**-4.48**
**FAM83D**	family with sequence similarity 83, member D	**-4.49**
**CDC20**	cell division cycle 20 homolog (S. cerevisiae)	**-4.50**
**TAGLN3**	transgelin 3	**-4.91**
**NEFM**	neurofilament, medium polypeptide 150kDa	**-6.00**

Fold changes are for comparison between MSCs harvested at high cell confluence (~90%) and at low cell confluence (~50%). Positive values indicate higher and negative values indicate lower expression in MSCs harvested at high cell density. CC1, culture condition 1; CC3, culture condition 3. $p < 0.05$.
